# Review: Endophytic microbes and their potential applications in crop management

**DOI:** 10.1002/ps.5527

**Published:** 2019-07-27

**Authors:** James F White, Kathryn L Kingsley, Qiuwei Zhang, Rajan Verma, Nkolika Obi, Sofia Dvinskikh, Matthew T Elmore, Satish K Verma, Surendra K Gond, Kurt P Kowalski

**Affiliations:** ^1^ Department of Plant Biology Rutgers University New Brunswick NJ USA; ^2^ Centre of Advanced Study in Botany Banaras Hindu University Varanasi India; ^3^ U.S. Geological Survey Great Lakes Science Center Cleveland OH USA

**Keywords:** biostimulants, bacteria, endophytic microbes, fungi, microbiome, rhizophagy cycle

## Abstract

Endophytes are microbes (mostly bacteria and fungi) present asymptomatically in plants. Endophytic microbes are often functional in that they may carry nutrients from the soil into plants, modulate plant development, increase stress tolerance of plants, suppress virulence in pathogens, increase disease resistance in plants, and suppress development of competitor plant species. Endophytic microbes have been shown to: (i) obtain nutrients in soils and transfer nutrients to plants in the rhizophagy cycle and other nutrient‐transfer symbioses; (ii) increase plant growth and development; (iii) reduce oxidative stress of hosts; (iv) protect plants from disease; (v) deter feeding by herbivores; and (vi) suppress growth of competitor plant species. Because of the effective functions of endophytic microbes, we suggest that endophytic microbes may significantly reduce use of agrochemicals (fertilizers, fungicides, insecticides, and herbicides) in the cultivation of crop plants. The loss of endophytic microbes from crop plants during domestication and long‐term cultivation could be remedied by transfer of endophytes from wild relatives of crops to crop species. Increasing atmospheric carbon dioxide levels could reduce the efficiency of the rhizophagy cycle due to repression of reactive oxygen used to extract nutrients from microbes in roots. © 2019 The Authors. *Pest Management Science* published by John Wiley & Sons Ltd on behalf of Society of Chemical Industry.

## 1 INTRODUCTION

### 1.1 Overview of endophytism in plants

An endophyte is any microbe (typically fungal or bacterial) that inhabits internal tissues of plants without causing disease.^1^, ^2^ All or most plants possess endophytes, and in most cases endophytes are seed transmitted and begin to promote growth and plant health as soon as seeds germinate.^3^, ^4^ Other endophytes may be recruited from the soil but similarly benefit plants.^4^ Endophytic microbes are important components of plants, and they function in the following ways: (i) increase nutrients acquired by plants,^5^, ^6^, ^7^, ^8^ (ii) defend plants from pathogens and insects,^9^, ^10^, ^11^ (iii) increase stress tolerance in plants,^12^, ^13^ (iv) modulate plant development,^13^, ^14^, ^15^ and (v) suppress weed growth.^16^ The particular mechanisms by which endophytic microbes fill the various functions in plants likely differ depending on the microbe and plant.^16^ In this review, we discuss the functions of endophytes, the mechanisms of activities of endophytes, and current and future applications of endophytic microbes in crops.

## 2 EFFECTS OF MICROBES ON PLANT GROWTH

### 2.1 Endophytes modulate plant development

Modulation of seedling development by endophytes is likely the result of the evolution of plants in continuous symbiosis with microbes that colonize plant tissues and thus reliably participate in the development process. The widespread capacity of many microbes to produce plant signal molecules (such as nitric oxide) growth regulators (such as auxins and ethylene) could be another reflection of co‐evolutionary association of microbes and plants. Controlled experiments revealed that seedlings of grasses cleaned of most of their endophytic microbes lose the root gravitropic response (*i.e*., roots do not grow downward), and seedlings frequently are diminished in size with reduced or no root hair formation.^14^, ^15^ The re‐inoculation of axenic or near axenic seedlings with microbes that internally colonize seedlings results in reacquisition of gravitropic response of roots and increased plant stature and root hair development.^14^, ^15^ Several experiments suggest that root hairs elongate until all microbes have been ejected from hairs.^21^ Root hair elongation may be triggered by nitric oxide or ethylene production by the intracellular microbe protoplasts that cluster in the tip of the elongating hair, but this has not been proven.^21^ It is unknown what is produced or degraded by the intracellular microbes to initiate the gravitropic response in seedling roots. Endophytic microbes in plants have also been shown to enhance root growth and increase root branching, further leading to increased plant growth.^1^, ^2^, ^13^ These effects of endophytes on root growth are generally attributed to production of growth regulators by microbes; however, enhanced nutrient acquisition from microbes may equally contribute to enhanced plant growth.

## 3 NUTRITIONAL FUNCTIONS OF ENDOPHYTES

### 3.1 Nutrient transfer symbioses

Endophytic microbes that bring nutrients to plants include those that fix atmospheric nitrogen in plant tissues. These types of endophytes include actinorhizal and rhizobial symbioses.^17^, ^18^ Because of sensitivity of nitrogenases to oxygen, the few families of plants that engage in nitrogen‐fixing symbioses sequester microbes in low oxygen nodules—where nitrogen is fixed and transferred to root tissues.^18^ Another type of nutritional endophytic symbiosis involves microbes that inhabit both endophytic tissues and extend out into soil. Dark septate endophytes and mycorrhizal fungi establish this kind of symbiosis with many families of plants. Hyphae of these fungi grow endophytically in roots, and the mycelia extending into soil acquire nutrients and mobilize it back to plants.^19^ Another nutrient acquisition mechanism involves the liberation of nutrients from insects by microbes that extend between, or cycle between, plants and decaying insects.^20^ In this process insects consume plants, accumulating nitrogen and other nutrients in their bodies; degradation of insects by microbes that are also symbiotic with plants results in transfer of nutrients to plants.^20^

### 3.2 Rhizophagy cycle and nutrient acquisition

**Figure 1:**
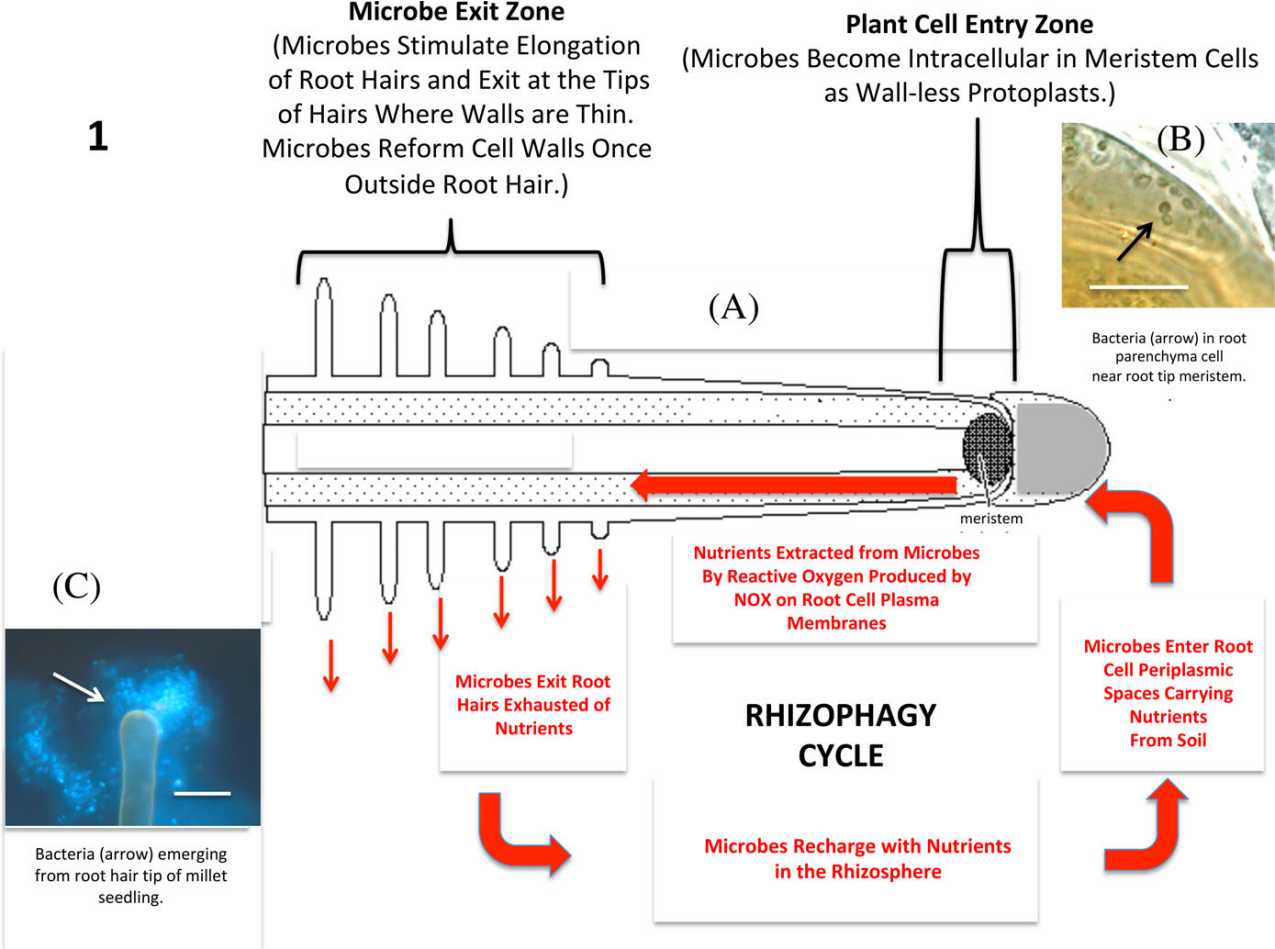
Diagrammatic representation of the rhizophagy cycle. (A) Diagram of the rhizophagy cycle showing microbes entering root cells at the root tip meristem and exiting root cells at the tips of elongating root hairs. Rhizophagy cycle microbes alternate between an intracellular endophytic phase and a free‐living soils phase; soil nutrients are acquired in the free‐living soil phase and extracted oxidatively in the intracellular endophytic phase. (B) Shows bacteria (arrow) in the periplasmic space of parenchyma cell near root tip meristem of an Agave sp. seedling (bar = 20 μm; stained with DAB followed by aniline blue). (C) Bacteria (arrow) emerging from root hair tip of grass seedling (bar = 20 μm; stained with fluorescent nucleic stain SYTO 9). Figure from *Microorganisms* 6 (3): 95. https://doi.org/10.3390/microorganisms6030095. (2018).

**Figure 2:**
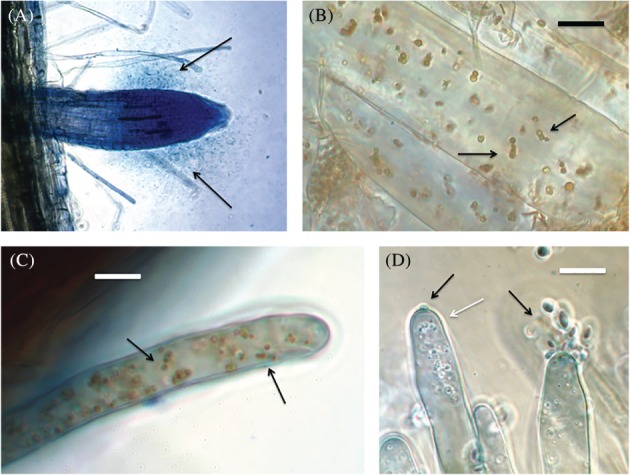
Microbes in plant roots. (A) Cloud of bacteria (arrows) around root tip meristem of invasive reed grass (*Phragmites australis*); stained with aniline blue (0.1% aqueous). (B) Root parenchyma cell of *P. australi*s showing replicating protoplasts of bacteria (arrows) in the periplasmic space of the cell (stained with DAB, followed by aniline blue; bar = 15 μ). (C) Root hair of grass *Cynodon dactylon* inoculated with bacterium *Pseudomonas* sp. showing bacterial protoplasts (arrows) in the periplasmic space of the hair (stained with DAB, followed by aniline blue; bar = 15 μ). (D) Root hairs of clover (*Trifolium repens*) inoculated with endophytic yeast (*Rhodotorula* sp.) showing yeast being expelled from the root hair tips (black arrows) and yeast protoplasts within root hairs (white arrow) (stained with DAB, followed by aniline blue; bar = 15 μ).

In another symbiosis, the ‘rhizophagy cycle’ or ‘rhizophagy symbiosis’, microbes (often bacteria or yeasts) cycle between an endophytic/intracellular protoplast phase in root cells and a free‐living walled phase in the soil (Fig. 1)‐(C).^21^ Microbes acquire nutrients in the free‐living soil phase and nutrients are oxidatively extracted from microbes in the endophytic/intracellular protoplast phase.^21^ Any nitrogen fixation by microbes involved in the rhizophagy cycle likely occurs in the free‐living soil phase because high levels of reactive oxygen secreted from root cell plasma membranes onto microbes inhibit nitrogenases.^21^ In the rhizophagy cycle, microbes are provided nutrients by plants *via* root exudates (*e.g*., carbohydrates, proteins, vitamins, organic acids) around the root tip meristem (Fig. 2)).^21^ Microbes are internalized into meristematic root cells that do not have fully formed cell walls just beneath the exudate zone.^21^ The mechanism by which microbes are internalized into root cells is unknown. However, once internalized microbes become situated in the periplasmic space, between cell wall and plasma membrane of root cells, where root cells secrete superoxide produced by root cell plasma membrane bound NADPH oxidase (Nicotinamide Adenine Dinucleotide Phosphate Oxidase) onto microbes (Fig. 2(B)).^21^, ^22^ Exposure to reactive oxygen (superoxide) produced by root cells triggers the loss of cell walls by the intracellular microbes; bacteria form protoplast phases called ‘L‐forms’, while fungi form protoplast phases termed ‘mycosomes’.^23^ This protoplast phase may be compared to the bacteroids of rhizobia that are also microbe phases involved in nutrient exchange with host cells.^18^ Suppression of superoxide formation in plant roots using elevated carbon dioxide results in failure of microbes in root cells to convert to protoplast phases (J. White, R. Verma, N. Obi, Unpublished). Protoplast forms of microbes ‘bud’ or ‘bleb’ sequentially in root cells as they replicate with older microbe cells/protoplasts often being oxidized completely—swelling and disappearing as cells age (Fig. 2(B)).^23^ Microbe protoplasts are circulated rapidly (circulation estimated at 8–11 μ s^−1^) around the periphery of root cells through the action of cyclosis.^21^ Cyclosis of protoplasts results in increased replication of microbe protoplasts with many small protoplasts being formed from fewer original intracellular microbe cells. Exposure of intracellular bacterial protoplasts to reactive oxygen results in electrolyte leakage from bacterial protoplasts, and oxidized bacterial components may be absorbed through the plasma membrane by plant root cells.^21^ The constant circulation of microbes against the root cell plasma membrane may reduce nutrient gradients between microbe and root cell protoplasts, resulting in more efficient nutrient transfer between the two cells. Surviving intracellular microbes that accumulate in the root hair tip trigger the elongation of root hairs (perhaps by nitric oxide signaling) and microbe protoplasts are periodically forcibly ejected through pores that form in root hair tips—with microbes reforming their cell walls as they reenter soil populations in the rhizosphere (Fig. 2(C,D)).^21^ The stimulus that triggers root hairs to periodically eject microbe protoplasts is unknown. However, the likely mechanism of ejection may involve potassium loading into the vacuole at base of the hair cell with consequent expansion in the hair vacuole that propagates from the hair base to the tip, forcibly expelling microbe protoplasts from the hair.

In the rhizophagy cycle, plants cultivate microbes that function as carriers of nutrients and support plant growth. In one experiment it was found that grass plants obtained approximately 30% of nitrogen from rhizophagy.^24^, ^25^, ^26^ Gene responses of plants infected with endophytes often show that plant antioxidants and nitrate transporters are among genes upregulated in plants.^7^, ^8^, ^13^ These upregulated genes may be the result of increased oxidative reactions in roots and increased liberation of nitrates resulting from protein degradation. Another experiment involving tomato seedlings employed elevated carbon dioxide to suppress superoxide formation and extraction of nutrients from microbes; here rhizophagy cycle suppression resulted in reduced absorption into seedlings of potassium, calcium and sulfur (J. White, R. Verma, N. Obi, unpublished). However, it may be that acquisition of difficult to obtain micronutrients (*e.g*., iron, copper, zinc) is a key function of the rhizophagy cycle. Microbes possess siderophores and other mechanisms that sequester micronutrients efficiently and many are motile and move around in soils acquiring nutrients.^27^ Metals also adhere to the cell walls of microbes because microbe cell walls have a net negative charge, while metals have a positive charge.^27^ Unlike nodule‐forming symbioses, the rhizophagy cycle appears to occur in most vascular plants and likely represents an important mechanism for nutrient acquisition by plants.^27^

### 3.3 Plants use microbes to mine for soil metals

**Figure 3:**
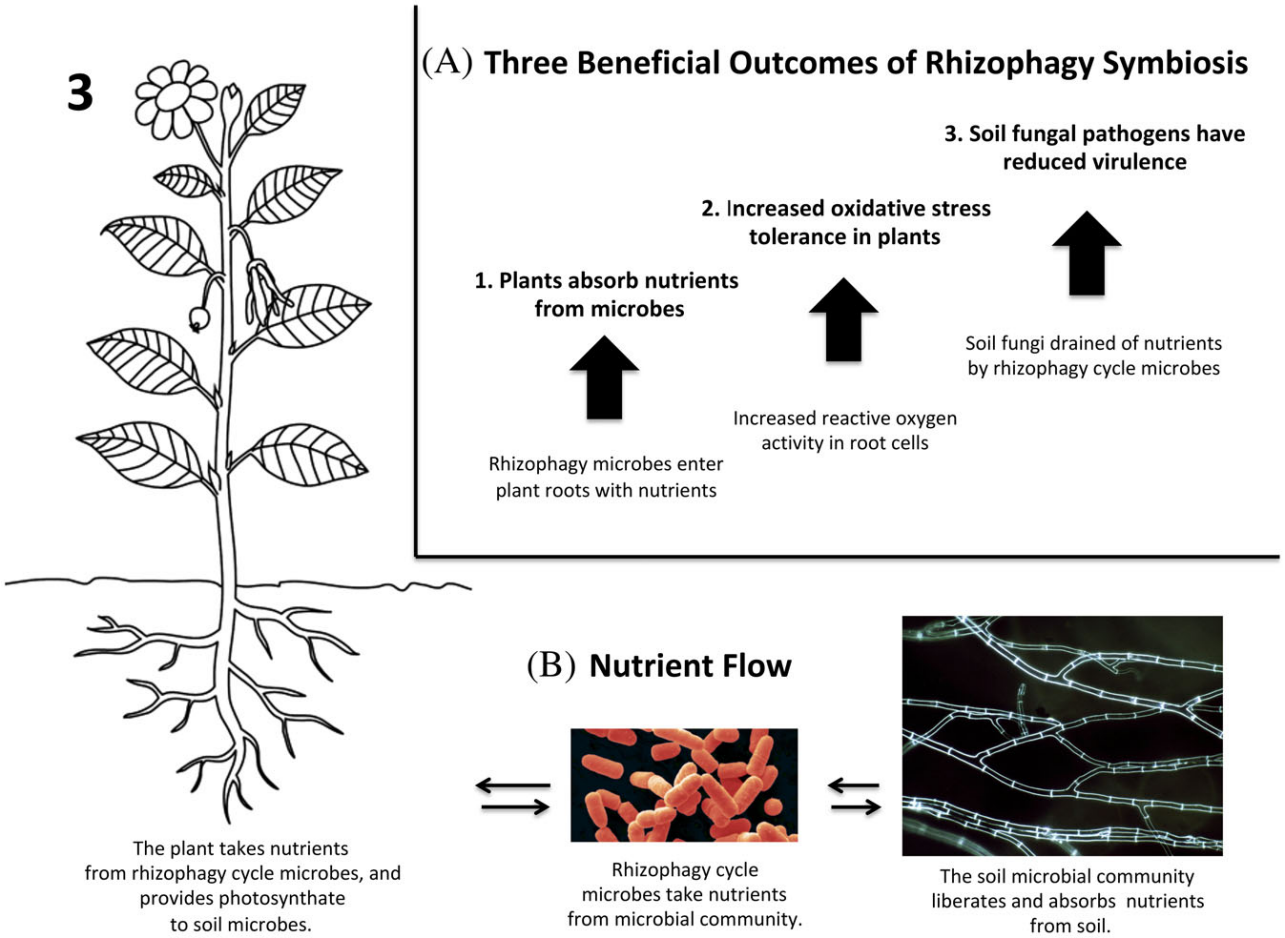
(A,B) Beneficial outcomes and nutrient flow in the rhizophagy symbiosis. (A) Beneficial outcomes include: (i) Plants obtain nutrients from internalized microbes; (ii) Increased production of reactive oxygen in roots results in increased oxidative stress tolerance in plants; and (iii) Scavenging of nutrients from soil fungi by rhizophagy microbes results in reduced virulence of potential pathogens in the soil microbial community. (B) In terms of nutrient flow, rhizophagy microbes mediate between the plant and the soil microbial community, with photosynthate and other plant‐abundant nutrients flowing from plant roots to soil microbial community; rhizophagy microbes carry nutrients from the soil microbial community back to the plant.

Plant roots secrete organic acids, including acetic acid, citric acid, and malic acid.^28^ These organic acids have a high affinity for metals, including iron, zinc, copper, and magnesium.^28^ Many microbes (*e.g*., *Bacillus* spp.) possess high affinity transporters that enable them to detect and absorb these organic acid‐metal complexes.^28^ Microbes benefit nutritionally by absorbing the organic acid‐metal complexes, in that they acquire carbon nutrients in the organic acids, and mineral nutrients simultaneously.^28^ The entry of the microbes into the root cells permits plants to extract the metals from the microbes. Harvesting of metals from the soil microbes *via* the rhizophagy cycle likely gives plants the critical soil nutrients needed for sustenance and growth (Fig. 3)).

### 3.4 Rhizophagy microbes take nutrients from other soil microbes

Rhizophagy microbes, such as *Bacillus* spp., have the capacity to extract nutrients from other soil microbes by causing nutrient leakage from their cells. This enables them to access nutrients contained in the soil microbial community and carry those nutrients back to the plant (Fig. 3(B)). Rhizophagy microbes take nutrients from other microbes using ‘hemolysins’ (biosurfactants) that form pores in microbe membranes, causing them to leak nutrients.^29^ *Bacillus* spp. frequently possess hemolysins that are lipopeptides, which act as biosurfactants that increase membrane porosity and induce nutrient leakage from affected cells, typically fungi.^30^ In the soil *Bacillus* spp. and some other bacteria colonize hyphae of soil fungi and use lipopeptides to induce leakage in the hyphal membranes.^4^, ^13^ This capacity of some microbes to tap into nutrients contained in soil microbes emphasizes the importance of a diverse and healthy soil microbial community. In a sense, plants integrate into the soil microbial community as soil nutrients and fixed nitrogen flow from soil microbes to plants and then photosynthate from plant roots flows out to the soil microbial community (Fig. 3B). Microbes involved in the rhizophagy symbiosis may also solubilize or make available other nutrients in the rhizosphere that are absorbed by the plant.^31^

## 4 CARBON DIOXIDE SUPPRESSION OF RHIZOPHAGY CYCLE

### 4.1 Rising atmospheric CO_2_ levels and reduced efficiency of the rhizophagy cycle

Recent research on the effects of rising atmospheric CO_2_ levels on nutrient content of major food crops shows an inverse relationship between CO_2_ level and the efficiency of nutrient extraction from soils.^32^ C‐3 photosynthesis pathway plants are particularly affected by high CO_2_ levels, having reduced content in nitrogen and minerals including magnesium, zinc, and iron.^32^ This effect of CO_2_ in reducing nutrient acquisition by plants may be explained by the suppressive effect of CO_2_ on NADPH oxidase involved in the rhizophagy cycle. As described above, reactive oxygen (primarily superoxide) in the rhizophagy cycle functions to extract nutrients from microbes that enter root cells.^21^ Carbon dioxide suppresses formation of superoxide needed to extract nutrients from microbes.^33^ Increasing the level of CO_2_ by 50% in air around seedlings of wheat, tomato, and tall fescue seedlings (with C‐3 photosynthesis pathway) substantially reduced the amount of reactive oxygen (superoxide) secreted by root cells onto microbes, resulting in fewer nutrients being extracted from intracellular microbes (J. White, R. Verma, N. Obi, unpublished). In C‐4 photosynthesis plants, CO_2_ is sequestered into 4‐carbon oxaloacetate and moved into bundle sheath cells thus is unavailable to inhibit reactive oxygen formation. If the suppressive effect of CO_2_ on nutrient extraction from microbes is supported in future experiments, then increasing levels of CO_2_ in the atmosphere may cause agricultural scientists to seek ways to neutralize the effect of CO_2_ on the rhizophagy cycle. Potential future solutions to counteract decreased efficiency of oxidative nutrient extraction from microbes could be: (1) the use of soil microbes that produce fewer antioxidants and are more susceptible to oxidative nutrient extraction; (2) the use of CO_2_ scrubbers to locally reduce CO_2_ in air to levels that are optimal for the rhizophagy cycle; (3) the development of techniques/technologies to increase oxygen levels around plants or plant roots to increase efficiency of reactive oxygen generation in the rhizophagy cycle; or (4) the development of plants that show increased root reactive oxygen secretion or reduced CO_2_ sensitivity. It is also possible that plants may evolve to become less sensitive to increasing atmospheric CO_2_ levels as is exhibited in plants with C‐4 photosynthesis.

## 5 APPLICATIONS OF ENDOPHYTES IN AGRICULTURE

### 5.1 Plant domestication and loss of endophytic microbes

Plants in natural communities maintain symbiotic associations with endophytic microbes that support growth and protect plants against biotic and abiotic stresses.^34^, ^35^, ^36^ However, symbiotic microbes may be lost during domestication and long‐term cultivation. In a cropping experiment using an annual wild tobacco (*Nicotiana attenuata)*, it was found that 7 years of continuous cultivation and seed cleaning, resulted in symbiotic microbe loss and increasing levels of disease due to fungal pathogens in genera *Fusarium* and *Alternaria*.^37^ Reacquisition of those microbes from wild populations of the tobacco and application to seedlings in cultivation resulted in resistance to the disease. Acid treatment of cotton seeds to remove fibers removed natural seed‐vectored microbes, leaving cotton seedlings more vulnerable to stress and disease.^38^ Acquisition of microbes from seeds of uncultivated plants in the cotton family greatly improved stress and disease resistance in cotton seedlings.^38^ The high levels of diseases and pests that plague cotton could be the result of the loss of symbiotic microbes from cotton seeds. Crops such as maize have been intensively cultivated and modified to the extent that external seed structures that vector microbes like hulls once present in ancestral teosinte have been lost.^36^ Modern hybrid maize varieties require higher inputs of nitrogen and pesticides to produce crops than older flint‐type Indian maize or tropical maize—and this may be the result of loss of symbiotic endophytes from hybrid maize varieties.^36^ Some grass seeds, such as Bermuda grass (*Cynodon dactylon*) are routinely cleaned of the microbial rich seed husk and covered with fungicides, leaving grass seedlings without their natural endophytes.^11^ We do not know the effects of long‐term use of inorganic fertilizers, fungicides, or other agrochemicals on endophytic microbes in crop plants and it is possible that long‐term agrochemical use has caused a loss of symbiotic endophytic microbes from many crop species. The loss of individual components of the natural endophytic community could alter how the seed community of microbes functions and result in seeds that are less capable of growth and survival. To remedy losses of essential endophytic microbes and reduce reliance on agrochemicals in crop cultivation, it could be necessary to obtain endophytic microbes from wild relatives of crops and reintroduce them into crops—perhaps as seed treatments.^15^

### 5.2 Mechanisms for endophyte‐mediated disease suppression

Among the many ways that endophytes improve plant health, one is by suppressing pathogen growth and fitness.^21^ This involves several mechanisms including direct antagonism by competition with pathogens for space and nutrients through production of antimicrobial metabolites and through induction of systemic resistance or increasing resistance in plants against pathogens *via* upregulation of host defense genes.^38^, ^39^ There are increasing numbers of studies that suggest that endophytes (fungi and bacteria) provide defense to host plants against pathogens and other pests beginning at seed germination and lasting the life of the plant.^39^, ^40^, ^41^ Bacterial endophytes of genus *Pseudomonas,* including *P. aeruginosa* and *P. fluorescens,* produce a variety of antifungal compounds, including phenazine‐1‐carboxylic acid, 2, 4‐diacetylphloroglucinol, pyrrolnitrin, pyoleutirin and volatiles like hydrogen cyanide compounds that significantly inhibit the growth of fungal pathogens.^41^, ^42^, ^43^ Species of genus *Bacillus* are important disease control agents because they synthesize a variety of biologically active molecules that are potential inhibitors of phytopathogens.^41^ A variety of lipopeptides that they produce induce leakage in fungal hyphal membranes that greatly reduces their virulence as pathogens of plants.^41^ This may result in a ‘quorum‐quenching effect where pathogenic fungi remain avirulent rather than causing disease. Many of the antifungal compounds produced by endophytes target membranes of fungi, inducing nutrient leakage, resulting in reduced virulence of the fungi.^41^, ^42^, ^43^ Endophytic symbionts also may improve plant resistance and protect plants against a broad spectrum of pathogens, particularly through induced systemic defense (ISR) by upregulating salicylic acid (SA) and jasmonate (JA) pathways and ethylene or PR proteins.^43^

### 5.3 Endophytes alter oxidative stress tolerance in plants

Environmental stresses trigger plant cells to form reactive oxygen species (ROS; including superoxide, hydroperoxyl radicals, hydrogen peroxide, and hydroxyl radicals).^44^ The release of ROS within plant tissues and cells can cause oxidative damage to plant proteins, nucleic acids, and membranes.^44^ Some endophytes induce stress tolerance to both biotic and abiotic stresses.^44^ At early stages of endophytic colonization, plant defense responses are activated, producing ROS.^44^ A q‐PCR analysis showed that bacteria at the early stages of colonization caused upregulated transcript levels of ROS‐degrading genes including superoxide dismutase and glutathione reductase.^44^ The upregulation of host ROS‐degrading genes may further reduce oxidative damage to plants by pathogens that induce or produce ROS. Tall fescue (*Festuca arundinacea*) grass tissues infected by the endophytic fungus *Epichloë coenophiala* have higher concentrations of osmoprotective mannitol and other antioxidant fungal carbohydrates involved in protection of plants under oxidative stress.^44^ Endophytic fungus *Piriformospora indica* has been shown to induce abiotic stress tolerance in many plants.^45^ *Piriformospora indica* infected Chinese cabbage (*Brassica rapa*) treated with polyethylene glycol to mimic drought stress, exhibited upregulation of antioxidant enzymes peroxidases, catalases, and superoxide dismutases in leaves within 24 h.^46^ The expression of drought‐protective genes *DREB2A*, *CBL1*, *RD29A* and *ANAC072* were upregulated in leaves of endophyte‐containing plants.^46^ A meta‐genome analysis of rice endophytes showed the presence of numerous genes encoding enzymes involved in protection from excessive ROS, including glutathione synthases and also glutathione‐S‐transferases.^47^ The endophytic bacterium *Enterobacter* sp. 638 isolated from stems of poplar trees was shown to have genes that encode several superoxide dismutases, including *SOD A*, *SOD B* and *SOD C*; in addition it possessed genes for catalases, hydroperoxide reductases, hydroperoxide reductases, and thiol peroxidases.^48^ The potent antioxidant compounds pestacin and isopestacin have been isolated from endophytic *Pestalotiopsis microspora*.^49^ Endophytes are also reported to reduce oxidative stress generated in plants in metal contaminated soils.^50^ The infection of soybean by endophytic *Paecilomyces formosu*s significantly reduced lipid peroxidation, and increased formation of peroxidase, polyphenol oxidase, catalase, and superoxide dismutase in Ni contaminated substrates.^51^

### 5.4 Endophyte‐mediated anti‐herbivory

Some endophytes produce and fill plants with compounds that reduce herbivory by insects and other herbivores.^52^ Species of fungal endophytes in genus *Epichloë* (Clavicipitaceae) intercellularly inhabit aerial parts of plants (*i.e*., leaves, culms, and seeds) and produce a variety of alkaloids that deter feeding by herbivores.^52^ These endophytes have found application in increasing pest tolerance in commercial forage and turf grasses. However, endophytes of this group of organisms are limited to grasses and sedges. Fungal endophytes (genus *Periglandula*) in the morning glory family (Convolvulaceae) have been also shown to produce ergot alkaloids that make morning glories highly toxic to herbivores.^52^ Similarly, in plants commonly referred to as ‘locoweeds’ in the family Fabaceae, endophytic fungi in genus *Undifilum* (Pleosporaceae) produce the toxic alkaloid swainsonine, a powerful anti‐herbivore compound and toxin.^52^ These few examples suggest that endophytes that deter feeding by insect pests may be more common than has been currently documented. A more thorough examination of fungal and bacterial endophytes in plants may result in numerous additional endophytes that may be used in crops to reduce insect pest feeding or improve plant tolerance to feeding.

### 5.5 Transgenic endophytes

Transgenically modifying endophyte genomes could be a useful strategy and an alternative to genetic manipulation of the host plant.^53^ Genes introduced into endophytic microbes could confer new characteristics, which may be useful in bio‐control of plant pathogens, growth promotion of host plants, and/or production of medicines for humans or animals. For example, the endophytic bacterium *Clavibacter xyli* subsp. *cynodontis*, which colonizes the xylem of several plant species, was transgenically modified to express the *Bacillus thuringiensis* gene encoding endotoxin for control of insects.^54^ In another example, an endophytic *Burkholderia pyrrocinia* JK‐SH007 was transformed with the Bt endotoxin gene to express the insecticidal protein against the second stage of *Bombyx mori* instar silkworms.^53^ Further, an endophytic *Pseudomonas putida* WCS358r was modified with an antifungal gene and introduced into wheat with a resultant reduction in fungal populations in soil, including pathogenic *Fusarium* spp.^53^ It seems likely that future efforts to manage crops could involve the exploitation of transgenically modified endophytes. However, due to the mobility of endophytic microbes containment of the microbes to specific plants may be difficult or impossible.

### 5.6 Endobiome interference as a strategy to reduce weed growth

The symbiotic relationships between a host plant and its endophytic microbes are unique, which can become a liability for the plant.^55^ When introduced into plants other than their adapted host, some endophytic microbes can cause growth repression and death in seedlings.^56^ ‘Endobiome interference’ occurs where entry of non‐adapted microbial endophytes into plant cells and tissues results in repressed plant growth and disruption of functions of the endophyte‐host symbiosis.^56^ In a case of endobiome interference, a fungal endophyte (*Aureobasidium pullulans*), isolated from roots of a weedy yet native species *Froelichia gracilis* (Amaranthaceae), was introduced by seedling inoculation into the cells and tissues of seedling roots of the exotic plant species *Amaranthus hypochondriacus*, resulting in growth repression of the seedlings.^56^ Further, the bacterial endophyte *Micrococcus luteus*, originally isolated from tomato seeds and seedlings, was transferred to seedlings where they entered into seedling root cells of multiple plant species (including *Phragmites australis*, *Poa annua*, *Fallopia japonica*, *Rumex crispus,* and *Taraxacum officionale)*, reducing native endophytic bacteria and reducing seedling growth. Endobiome interference could be a common phenomenon in natural plant communities and could be one way that plants reduce growth of competitor plants. Similarly, if used as a management treatment, endobiome interference may have potential to reduce the invasive character of invasive and weedy plant species.^57^ Recently, an agenda was developed to evaluate how the microbial community could be targeted as a form of control for non‐native *Phragmites australis* (common reed), a strategy that could be adapted for other invasive and weedy plant species.^57^ The symbiotic relationships with its endophytes give the non‐native *P. australis* a competitive edge over native species,^57^ but the location and roles of individual microbes within the plant are still unclear.^57^

### 5.7 Hindrances and advances in applications of endophytes in agriculture

The lack of an overall awareness of the general presence of communities of endophytic microbes in tissues of plants has been a hindrance in advancing exploration of applications of endophytes in crops. Add to this, the general and prevalent assumption that most microbes on plants are pathogenic or have negligible effects has contributed to lack of efforts to understand the roles of endophytes in plant growth promotion and plant health improvement. Over the past few decades the gradual development of a body of research that demonstrated that endophytes are common in plants and positively affect plant development and health represents an important advancement in understanding the importance and functionality of endophytes.^1^, ^7^, ^8^, ^35^, ^45^, ^46^, ^58^ Recently, the exploration for useful endophytes and other microbes for agricultural applications has been enhanced by emergence of companies that have as their primary focus development and marketing of plant biostimulants (including endophytes).^59^ To facilitate development of applications for microbes in agriculture, the recent U.S. government 2018 Agriculture Improvement Act (also known as the 2018 Farm Bill) in the United States and regulatory documents in the European Union explicitly addressed biostimulants and their regulation.^59^ The scientific, legal and regulatory framework appears to be in place for some significant future advancements in products and applications of endophytic microbes in agriculture.

## 6 CONCLUSIONS

Microbial endophytes and soil microbes could be employed to improve plant health and enhance productivity directly in commercial crop plants. Benefits could also be realized when endophytes reduce pathogens, insect damage, and competition with weedy plants. Increasing crop productivity without harming the health of agricultural soils and compromising food quality with agrochemicals can be a challenge using present agricultural practices. The current efforts to find microbial crop stimulants are a beginning that may lead to a significant reduction in chemical applications in crop production. Endophytes could help cultivate crops with less fertilizers, fungicides, insecticides, or herbicides. In the future, we envision a change in practice to focus more on the optimization of the relationship of plants to soil microbes and endophytes. Supplementing microbial diversity through microbe amendments to soils and plants that function to bring nutrients to plants (*e.g*., through the rhizophagy cycle), while simultaneously suppressing virulence in pathogens, deterring insect feeding, and reducing growth of competitor weeds, can result in less environmental contamination and agricultural practices that are more parsimonious with natural processes. To bring about this future, we must develop a better understanding of how microbes function in soils and in plants. We must further learn how to optimize microbial functions to enhance crop production and protection.
